# Rectosigmoid endometriosis: Diagnostic pitfalls and management – A case report

**DOI:** 10.1002/ccr3.5222

**Published:** 2022-02-20

**Authors:** Athanasios Piachas, Panagiotis Smyrnis, Andreas Tooulias

**Affiliations:** ^1^ Department of Surgery Papageorgiou General Hospital Aristotle University of Thessaloniki Thessaloniki Greece

**Keywords:** endometriosis, hemafecia, low gastrointestinal bleeding, rectosigmoid colon

## Abstract

Endometriosis constitutes a benign condition, occurring in 10%–12% of menstruating women. Bowel involvement is estimated to occur in 5%–12% with the rectosigmoid region involved in up to 90% of these cases. We present the case of a 45‐year‐old Caucasian female patient with rectosigmoid endometriosis.

## Graphical abstract

While endometriosis is a difficult radiologic diagnosis to make, it must be considered in women being worked up for a colonic mass. Biopsy is usually necessary to confirm the diagnosis, as endometriosis can mimic colonic cancer during initial work‐up.

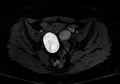

## 1 INTRODUCTION

Endometriosis refers to the presence of endometrial glands outside the uterine cavity.^1^ It is a benign gynecologic condition, occurring in 10%–12% of menstruating women.^1^ Endometriosis can manifest as ovarian, superficial peritoneal, or as deep infiltrating endometriosis (DIE).^2^ DIE lesions may affect the uterosacral ligaments, the rectovaginal space, and the gastrointestinal or urinary tract.^2^ Bowel involvement is estimated to occur in 5%–12% of women with endometriosis, with the rectosigmoid region involved in up to 90% of these cases.^1^

Rectosigmoid endometriosis may manifest with a variety of non‐specific symptoms, such as abdomino‐pelvic pain, alterations in bowel habits, or dyschezia.^3^ In rare cases, though, it can cause hemafecia, resembling the clinical profile of colorectal malignancy.^4^ Differential diagnosis from colorectal cancer may be quite difficult due to similar colonoscopic and radiologic findings.

Herein, we report an uncommon case of intestinal endometriosis, where the initial diagnostic work‐up indicated a colorectal malignancy. Our presentation demonstrates the role of clinical suspicion in achieving a timely preoperative diagnosis and consequently ensuring better outcome for the patient.

## 2 CASE PRESENTATION

**FIGURE 1:**
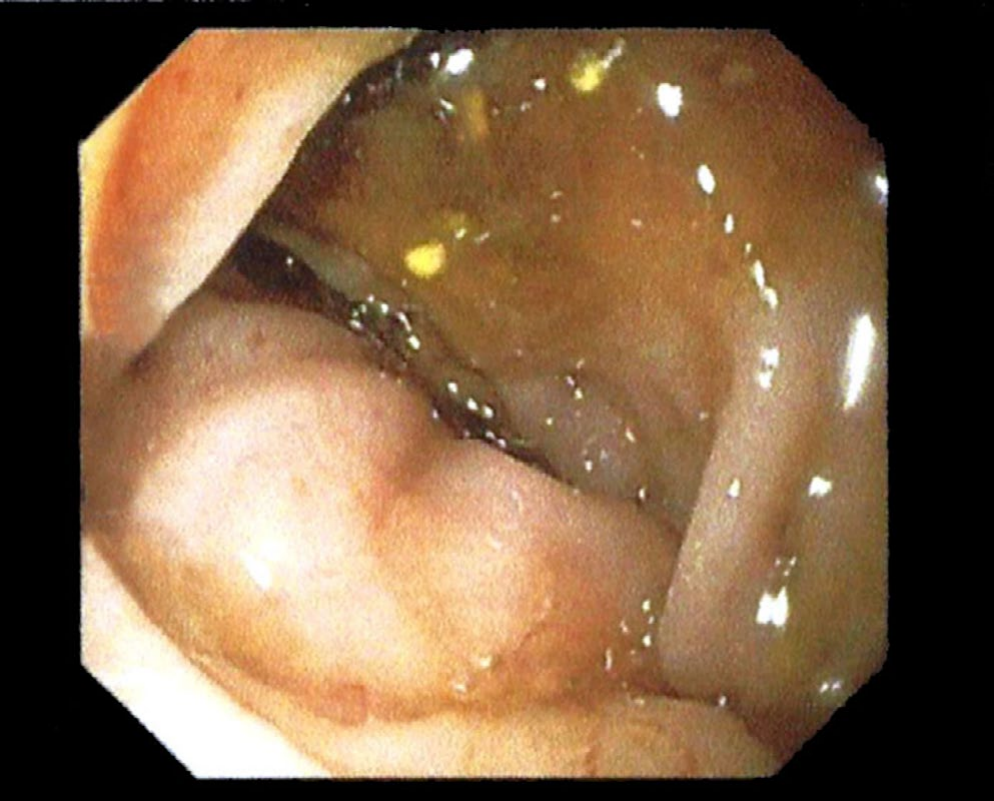
Colonoscopy image showing revealed a 5‐cm stricture of the sigmoid colon, located 25 cm above the anal ring

**FIGURE 2:**
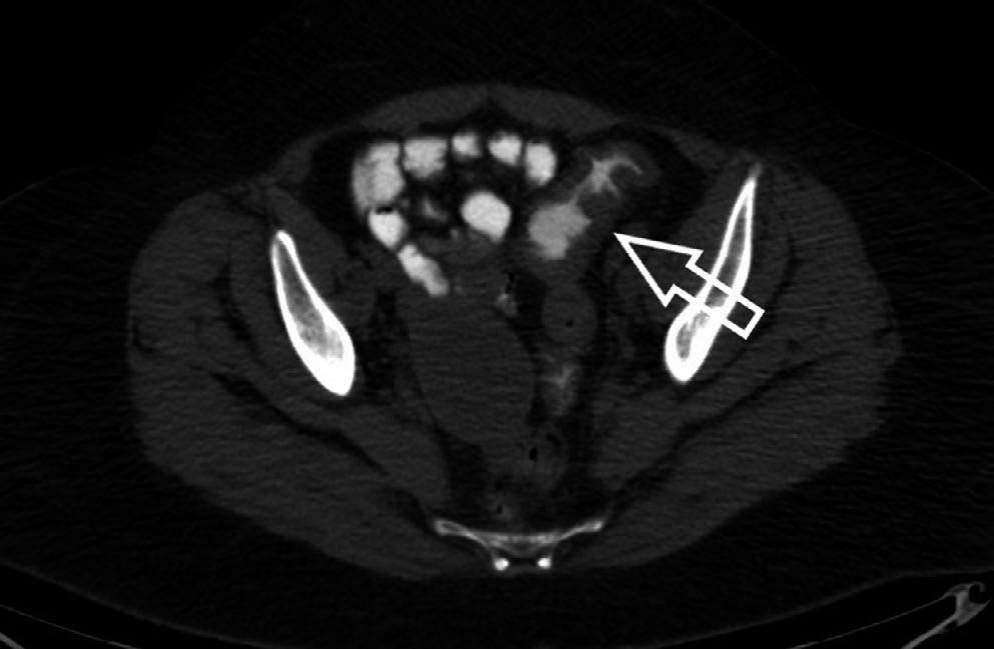
CT scan disclosed symmetric thickening of proximal sigmoid colon with a wall thickness of 2 cm (white arrow). It also detected a cystic lesion in the right ovary

**FIGURE 3:**
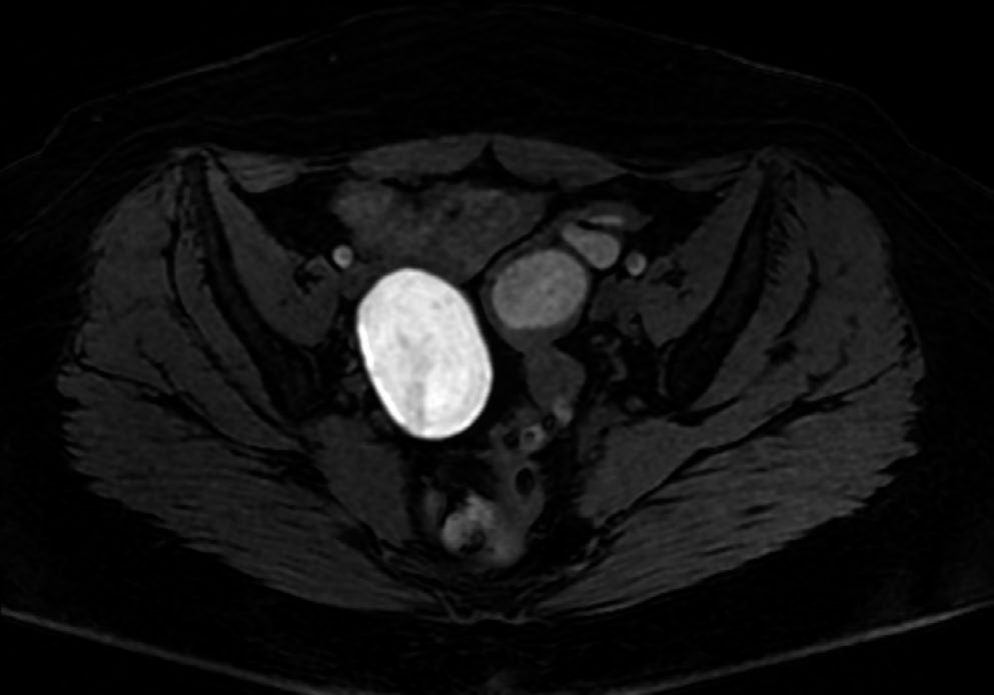
High T1‐weighted signal intensity of the ovarian cyst demonstrated the hemorrhagic content of the lesion

**FIGURE 4:**
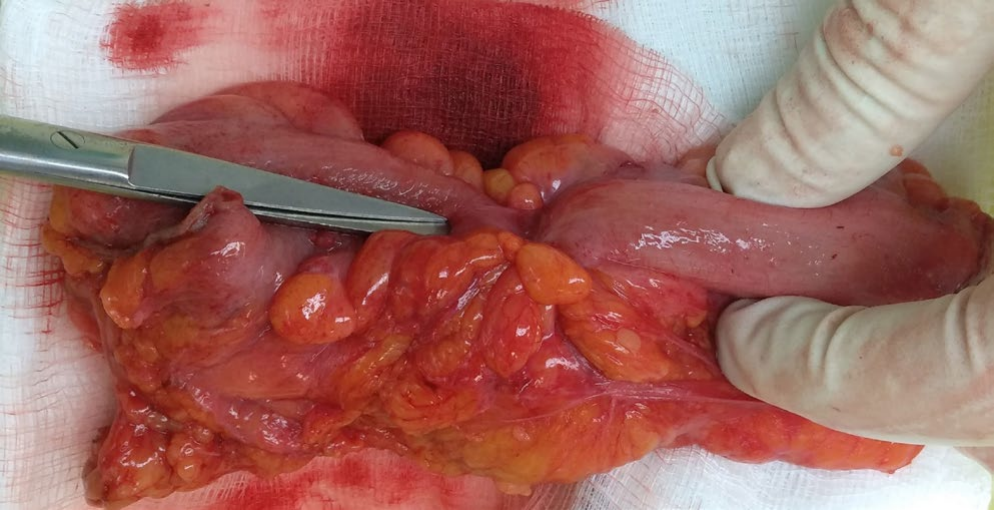
Gross appearance of the resected specimen with a visible endometrial implant on the serosal surface

**FIGURE 5:**
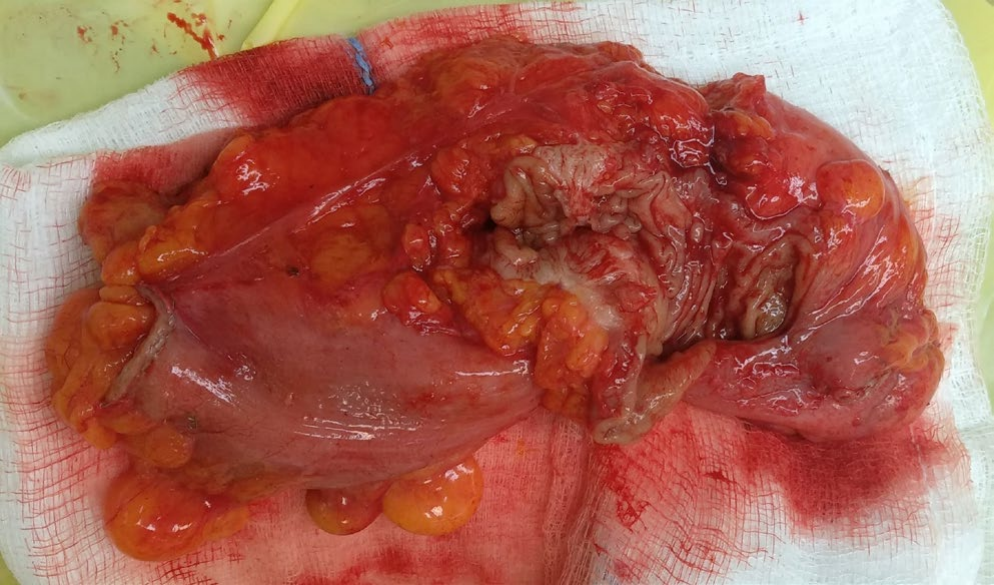
Cross section through the bowel wall reveals a predominant fibrous mural mass resulting in a stricture

**FIGURE 6:**
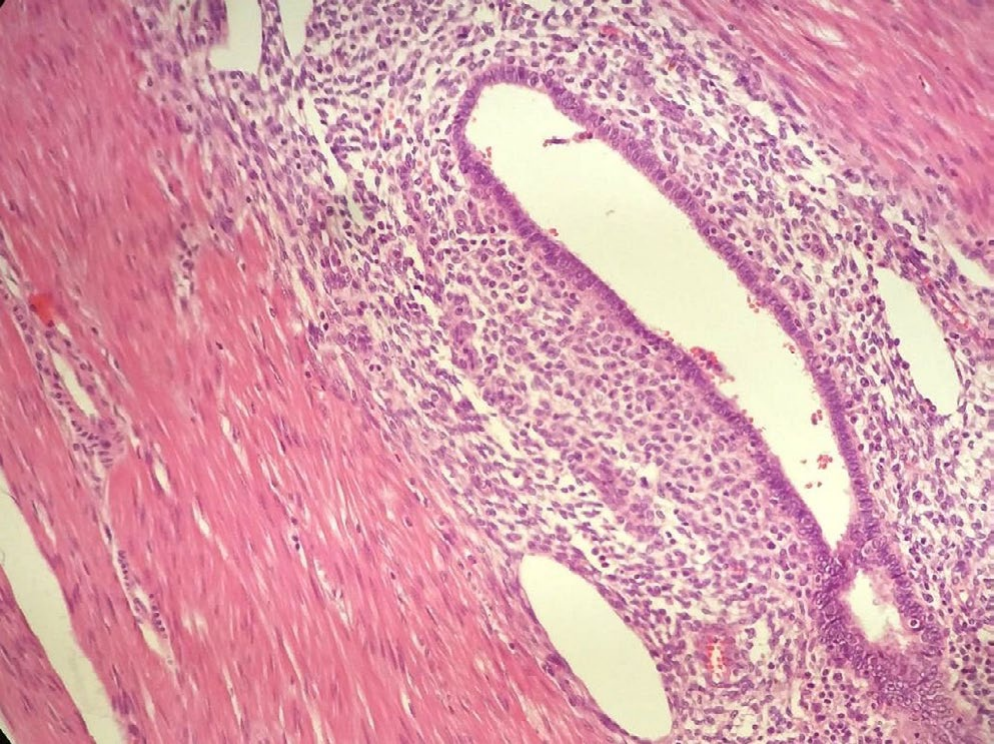
Biopsy of the resected colon revealed the presence of transmural endometrial glands surrounded by stroma

A 45‐year‐old Caucasian female patient presented to the emergency department complaining of recurrent episodes of hematochezia. She had been also suffering from constipation and mild left‐lower quadrant abdominal pain for the last 6 months. Regarding her medical history, the patient was perimenopausal and on treatment with low dose ethinylestradiol/chlormadinone. Furthermore, she was treated with amlodipine because of arterial hypertension. The patient also reported a familial history of ulcerative colitis including her sister (Figures 1, 2, 3, 4, 5, 6).

The patient had been recently hospitalized due to severe diarrhea, which shifted into rectal bleeding. Initial diagnosis was gastroenteritis, therefore she was treated conservatively with IV administration of ciprofloxacin and metronidazole. No colonoscopy was performed during her hospitalization. She was discharged after 5 days and was fully recovered.

On physical examination, the patient presented with normal vital signs. Abdominal examination disclosed a soft, non‐distended abdomen with left‐lower quadrant tenderness. No palpable masses were present. Digital rectal examination revealed the presence of bright red blood. Laboratory evaluation demonstrated hemoglobin of 11.4 g/dl with normal WBC count. Electrolytes, renal function, and liver enzymes were within normal values. Initially, the patient was managed with intravenous fluid resuscitation. Nasogastric aspiration revealed no signs of active bleeding in the upper gastrointestinal tract. Subsequently, she was subjected to a colonoscopy. This disclosed a 5‐cm stricture of the sigmoid colon, located 25 cm above the anal ring.^1^ In order to elucidate the lesion, a computed tomography (CT) of the abdomen and pelvis was performed. It revealed symmetric thickening of the proximal sigmoid colon, with a wall thickness of 2 cm, along with surrounding fat stranding.^2^ It also detected a cystic lesion in the right ovary, measuring 6 cm in diameter with a slightly high density (30–40 HU).^3^ Malignancy could not be ruled out, thus serum tumor markers were also ordered. AFP, CEA, and CA19‐9 were all within normal limits.

The patient was scheduled for an open surgical resection. Intraoperatively, a structuring lesion was distinguished in the distal sigmoid. Furthermore, an ovarian chocolate cyst was identified in the right ovary. The patient underwent a sigmoidectomy followed by an end‐to‐end anastomosis, combined with right salpingo‐oophorectomy.

Gross examination of the resected specimen disclosed a fibrous transmural mass along with endometrial implants on the serosal surface.^4^, ^5^ Subsequent histopathologic examination revealed the presence of endometrial glands surrounded by stroma, refining the diagnosis of sigmoid endometriosis. No malignant lesions were detected. Moreover, microscopic evaluation of the ovarian specimen also confirmed the endometrial lesion.^6^

## 3 DISCUSSION

Clinical presentation of intestinal endometriosis is characterized by non‐specific symptoms with considerable overlap with other clinical entities.^5^ A detailed medical history along with a thorough physical examination remains crucial for an accurate and timely diagnosis. Clinical manifestations vary depending on the location and the infiltration depth of the lesion. Nevertheless, chronic pelvic pain represents the most commonly described symptom. It may be experienced as dysmenorrhea, deep dyspareunia, or even as non‐menstrual pelvic pain.^6^ Deeply infiltrative lesions of the muscularis may present with miscellaneous gastrointestinal symptoms, such as abdominal pain, constipation, diarrhea, abdominal distension, and occasionally tenesmus.^1^ Rectal bleeding remains quite rare, since the mucosa is rarely infiltrated by endometrial nodules.^7^ Rectovaginal examination at the time of menstruation is considered quite helpful, since endometrial lesions may be more inflamed or tendered.^8^ Notable findings include palpable nodules along the region of uterus, uterosacral ligaments, or rectovaginal septum.^8^

Several imaging techniques are quite conducive to diagnosing intestinal endometriosis. Transvaginal ultrasonography is characterized by high sensitivity and specificity, but it may miss sigmoid lesions, since the latter remain outside of field of vision.^9^ MRI is a less operator‐dependent technique and better suited to identify lesions located above the rectosigmoid junction.^9^ It lacks sensitivity for defining the depth of infiltration, since bowel peristalsis may cause artifacts.^8^ In addition, there are still lesions that might be missed due to their fibrotic component.^10^ Colonoscopy remains of low value in the diagnosis of endometriosis, since endometrial lesions are typically extrinsically, and thus, not visible during the examination.^5^ Furthermore, mucosal biopsy is often unhelpful, since the lesions are limited to the serosa.^11^ Other modalities such as CT, barium enema, or endorectal ultrasound have been studied, yet with mixed outcomes.^8^ The gold standard to confirm the diagnosis is still direct visualization with laparoscopy.^11^

Surgical resection and conservative treatment are the two major management strategies widely adopted, once bowel endometriosis is diagnosed.^12^ Medical management should be considered primarily in women who are not surgical candidates and those who are not interested in immediate pregnancy.^8^ Oral contraceptives (OC) and progestins constitute the first‐line therapy.^13^ The resultant pseudo‐menopausal status reduces the fluctuations of gonadal steroids, stimulating atrophy of endometrial lesions.^14^ Gonadotropin‐releasing agonists or danazole remain secondary option.^15^ Nevertheless, hormonal agents are considered to be suppressive rather than curative, since they do not have an impact on the meta‐inflammatory fibrotic component.^16^ Furthermore, it should be administered continuously for a long‐term period, since symptoms are about to relapse once treatment is ceased.^16^ Consequently, it constitutes symptomatic treatment.

Surgical intervention is advisable in patients with symptomatic lesions refractory to medical treatment, obstructive disease, and exclusion of malignancy.^17^ It includes nodule excision and colorectal resection. Nodule excision may be performed either without opening the intestinal wall (shaving excision) or by concomitant removal of the surrounding bowel wall (discoid excision).^18^, ^19^, ^20^

Shave excision represents the least invasive technique, involving a layer‐by‐layer removal of the lesion. It may be performed either by ablation or resection of endometrial nodules, leaving muscularis and mucosa intact.^18^, ^19^ In order to be considered as a treatment option, there should be only superficial serosal lesions.^18^, ^19^ Discoid excision, on the other hand, implies a full‐thickness excision of the endometrial implant followed by primary closure of the resultant wall defect. Indication for such technique is solitary lesion encompassing less than half of the bowel circumference.^18^ Nevertheless, disk resection carries a high rate of postoperative complications.^8^

Segmental resection, eventually, entails complete resection of the affected bowel with subsequent anastomosis. As a more invasive type of surgery, it is indicated mainly for patients with bowel stenosis, multifocal lesions, sigmoid involvement, and lesions larger than 3 cm or involving >50% of the circumference of the bowel wall.^21^ Major postoperative complications include bowel denervation, loss of compliance, or hypersensitivity.^18^ Despite its higher rate of complications, segmental resection is associated with lower recurrence ratio compared with the above‐mentioned approaches.^18^

In conclusion, rectosigmoid endometriosis constitutes a rare cause of low gastrointestinal bleeding and also a major challenge for general surgeon, since the differential diagnosis from colorectal malignancy could be difficult. Representing a quite uncommon clinical entity, it should always be included in the differential diagnosis of hematochezia in women of childbearing age. Accurate and timely diagnosis remains crucial for an optimal therapeutic approach, highlighting the major role of a high clinical suspicion.

## CONFLICT OF INTEREST

None to declare.

## AUTHOR CONTRIBUTION

Athanasios Piachas reviewed the literature and the manuscript, made a contribution to drafting, and edited the images. Panagiotis Smyrnis reviewed the literature and wrote the manuscript. Andreas Tooulias reviewed the literature and the manuscript.

## ETHICAL APPROVAL

The authors declare that the current manuscript is not published elsewhere. This paper does not appear online, either wholly or in part, as a thesis, working paper series, or preprint publication.

## CONSENT

A written consent was obtained from the patient for publication of the case report according to the ethical principles of Declaration of Helsinki.

## Data Availability

All the authors confirm that the data supporting the findings of this paper are available within the article and/or its supplementary material.
